# Adoption of Biosecurity Practices in Smallholder Dairy Farms in Ethiopia

**DOI:** 10.1155/2023/2277409

**Published:** 2023-08-14

**Authors:** Ndungu S. Nyokabi, Stefan Berg, Adane Mihret, Gizat Almaw, Gizachew Gemechu Worku, Johanna F. Lindahl, James L. N. Wood, Henrietta L. Moore

**Affiliations:** ^1^Institute for Global Prosperity, University College London, London, UK; ^2^Bernhard Nocht Institute for Tropical Medicine, Hamburg, Germany; ^3^Armauer Hansen Research Institute (AHRI), Addis Ababa, Ethiopia; ^4^Animal Health Institute (AHI), Sebeta, Ethiopia; ^5^International Livestock Research Institute (ILRI), Nairobi, Kenya; ^6^Department of Veterinary Medicine, University of Cambridge, Cambridge, UK

## Abstract

Dairy production is an important livelihood source for smallholder dairy farmers who produce the majority of milk consumed and traded in Ethiopia. Dairy production is, however, constrained by livestock diseases that impact farm productivity, food safety, and animal welfare. Biosecurity measures (BSM) include all risk reduction strategies designed to avoid the introduction of pathogenic infections from outside and minimise the spread of diseases within dairy herds. This study used a cross-sectional survey to investigate the adoption of BSM in dairy farms in Addis Ababa and Oromia regions of Ethiopia. Using a questionnaire, scores for adopted external and internal BSM were calculated based on the Ghent's University Biocheck tool to compare the performance of different farms in Ethiopia. The weighted external biosecurity score was 49.1%, which was below average (below 50% adoption), while the weighted internal biosecurity score was 55.5%. Low adoption of crucial BSM increases the risk of disease introduction into dairy farms and transmission within herds. Adoption of BSM at the farm level was driven by individual, demographic, and socio-economic drivers, including education, farming system, milk value chain, and farming experience among others. Results of this research reveal low adoption of BSM and the imperative to encourage farmers to implement BSM can lead to a reduction in disease pressures and, thus, a reduction in antibiotic use and increased dairy farms productivity, and improved animal health and welfare. Farmers can be encouraged through proactive engagement with veterinarians and extension professionals. Moreover, creating a favourable policy environment can support farmers to adopt and implement BSM, given the known fact that “prevention is better and cheaper than curing diseases.”

## 1. Introduction

Dairy production in sub-Saharan Africa is an important livelihood source for smallholder dairy farmers who produce the bulk of the milk consumed and traded in the dairy value chains [1–3]. Milk is an important part of the diet in most communities globally and is a rich source of macro- and micro-nutrients needed for human well-being. Dairy production is, however, constrained by endemic zoonoses and livestock diseases, which impact farm productivity and animal welfare [2, 4]. Milk from smallholder farms contaminated with zoonotic microbial pathogens, including *Mycobacterium bovis*, *Escherichia coli*, and *Brucella* spp. pose a public health risk to consumers [5, 6]. Zoonoses cause morbidity, mortality, affect children, poor individuals and households, and immunocompromised individuals [4].

Globally, animal health management at the herd level is gradually shifting from cure-based towards disease prevention through the implementation of biosecurity measures (BSM) in dairy production processes [7]. BSM have been defined as “the implementation of a segregation, hygiene, or management procedure (excluding medically effective feed additives and preventive/curative treatment of animals) that specifically aims at reducing the probability of the introduction, establishment, survival, or spread of any potential pathogen to, within, or from a farm, operation or geographical area” [8]. BSM can be categorised as external and internal BSM [9]. External BSM are preventive and risk reduction strategies designed to avoid the introduction of pathogenic infections (hazards) from outside a farm. Internal BSM aim to prevent or limit within-farm transmission of infectious hazards, e.g., between cattle in a herd [8–10].

Viral and bacterial infectious agents causing diseases at farm level can be transmitted through several routes, mainly via aerosols and secretions at interactions between infected domestic or wild animals and non-infected livestock but also via fomites within a barn, including people, and possibly via trucks and other vehicles moving within and between farms [9]. Livestock diseases can negatively affect livestock production, animal welfare, and farmers' income, and diseases of zoonotic nature can impact the farmers' health, and they may also have a public health impact on the wider consumer population [11]. Implementing BSM for disease prevention can lead to many benefits, including improved livestock production efficiency, reduced livestock deaths, improved animal welfare, and good animal health, which positively influences immune response to vaccines. Moreover, these measures are likely to increase health and profits at farms in the long term, which leads to job satisfaction among producers, herd health professionals, and other agricultural workers [7, 9]. Additionally, the implementation of BSM has been shown to reduce the use of antibiotics on farms due to reduced disease pressure [9].

In the context of dairy production, BSM are the most cost-effective protection against cattle diseases [7, 9, 11]. However, the majority of dairy farmers rarely implement such measures on their farms, even though their benefits have been documented [10, 11]. Research studies have reported that the adoption and implementation of BSM in dairy farms are influenced by a number of factors, including access to information sources, social dynamics, official veterinary services, individual factors at farms, the individual experiences of farmers, availability of time and labour to implement, among other factors [10, 12].

In Ethiopia, the country with the largest cattle population in Africa, dairy production is an important source of livelihood for the majority of smallholder farmers [5, 6]. The most common breed of cattle in Ethiopia is the local zebu that is often kept in extensive grazing areas [2]. However, the majority of smallholder farmers are adopting exotic breeds and their crosses to increase milk production due to the growing demand driven by a growing population, increasing household incomes, and changing dietary patterns [3, 13]. However, these exotic and cross-bred cattle are usually more susceptible than the local breeds to endemic diseases such as lumpy skin disease [14], bovine tuberculosis [15, 16], and foot and mouth disease [17], leading to higher cattle mortality which constrains production and negatively impacts farmers livelihoods in the Ethiopian dairy production systems. Considering the high prevalence of livestock diseases, some of which are zoonoses of public health concern, it is worrying that there is a lack of research studies investigating the adoption and implementation of BSM for disease prevention in smallholder dairy farming systems. It is thus important to fill this research gap, considering the reported high prevalence of endemic zoonoses and livestock diseases, and the absence of livestock disease control programmes, to find a sustainable pathway to address animal health and public health challenges faced by smallholder farmers and consumers of milk and other dairy products. To the best of our knowledge, no study has investigated the adoption of BSM in Ethiopia. The aim of the study was, therefore, to assess the current status of farm-level adoption of BSM for the prevention of disease transmission within and between dairy farms in milk sheds of the capital Addis Ababa and its surrounding areas of the Oromia region in central Ethiopia.

## 2. Material and Methods

### 2.1. Study Area

**Figure 1:**
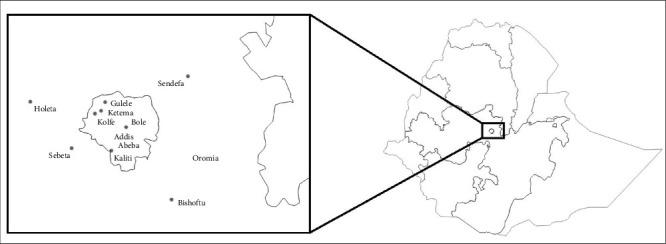
Map of the study area (author's own).

This research was conducted in April and May 2021 in central Ethiopia, in the wider Addis Ababa milk shed and its surrounding areas of the Oromia region (Figure 1). This milk shed is comprised of urban, peri-urban, and intermediate rural areas within a 60 km radius of Addis Ababa city, the capital of Ethiopia. These dairy production systems are important as they produce the bulk of milk sold through formal and informal dairy value chains in and around Addis Ababa. The urban study areas of Addis Ababa included Bole, Kolfte, Ketema, and Kaliti sub-cities, while the peri-urban study areas located in the Oromia region were made up of Sendafa, Sebeta, Debre Zeit, and Holeta towns.

### 2.2. Questionnaire Design

The questionnaire was designed based on biosecurity literature for cattle farms and publicly available biosecurity tools, including the Ghent's University biosecurity survey tool [18] and literature on cattle BSM by Sayers et al. [11] in Ireland, Shortall et al. [19] in the United Kingdom, Villaamil et al. [20] in Spain, Sarrazin et al. [7] in Belgium and Denis-Robichaud et al. [21] in Canada. The internal and external BSM were chosen based on the authors' literature review [2, 15, 22, 23], and the livestock disease risks specific to Ethiopia.

The selected BSM were categorised as external and internal BSM based on the risks they address at the farm level, as explained by Biocheck.UGent [18, 24, 25] and Sarrazin et al. [7]. The overall biosecurity score was the weighted sum of the measures. External BSM comprised a larger set of practices that were divided into five external biosecurity categories: livestock purchase and reproduction (eight practices), transport and carcase removal (seven practices), feed and water (eight practices), visitors and farmworkers (six practices), and vermin and pest control (six practices). These categories: livestock purchase and reproduction, transport and carcase removal, feed and water, visitors and farm workers, and vermin and pest control were weighted as 39%, 17%, 10%, 20%, and 14%, respectively, in their contribution towards disease prevention from outside of a farm as has been described by Biocheck.UGent [18, 24, 25] and Sarrazin et al. [7].

Internal BSM comprised another set of practices divided into six internal biosecurity categories: herd health management (eight practices), calving management (five practices), calves management (three practices), dairy management (eight practices), adult cattle management (five practices), and finally working organisation and equipment (five practices). The internal measures were weighted as 29%, 20%, 21%, 13%, 7%, and 10%, respectively, in their contribution towards herd-level disease prevention within a farm [7, 18, 24, 25].

The questionnaire used in this study was pretested in five farms outside the study areas, and adjustments were made to the chosen farm-level BSM. The questionnaire was administered by a team of trained enumerators who could speak both Amharic and Afaan-Oromo, the common languages in the study area.

### 2.3. Recruitment of Study Participants and Ethical Approvals

A total of 159 farmers were recruited through convenience and purposive sampling. These framers included participants from previous work by the Ethiopia Control of Bovine Tuberculosis Strategies (ETHICOBOTS) project. Dairy farm owners were briefed in the presence of a witness (local experts) on the study questionnaire that their participation in the study was voluntary and that confidentiality on survey results would be maintained before informed consent was obtained verbally. The research had Ethical clearance from the University College London Research Ethics Committee (UCL-REC) approval number 19867/001 and the Armauer Hansen Research Institute (AHRI) and ALERT hospital AHRI/ALERT Ethics Review Committee (AAERC) approval Protocol number PO-(46/14).

### 2.4. Measurement of Biosecurity

For each of the BSM, responses were recoded one if the biosecurity measure was adopted and 0 if it was not. The binary outcomes were then summed up to get the total number of measures per category and the mean calculated for each category of BSM (for both internal and external BSM). Furthermore, the sum of the binary outcomes was used as the dependent variable to run an ordinary least squares (OLS) regression model to explore the drivers of adoption of the various components of biosecurity as shown in Model 1. The independent variables used in the model were farmer education, cattle breed, labour availability, an extra source of income, and value chain choice among others.

(Model 1) $$\begin{array}{c} Y=\beta 0+\beta 1X1+\beta 2X2+\cdots +\beta pXp+\varepsilon , \end{array}$$

where *Y* is the sum of adopted measures in a category of internal or external BSM, *X* is the independent variables (i.e., farmer education, cattle breed, labour availability, an extra source of income, value chain choice, etc.) and *ε* is the error term.

Farm-weighted BSM were computed based on the formula developed by Biocheck.UGent [18, 24, 25] and Sarrazin et al. [7]. The formula for BSM for internal and external BSM considers the importance of the measures in the prevention of disease risks. The assigned weights add up to 100, and the BSM ranges between 0 and 100 (expressed as a percentage). BSM were computed as shown in Equation (1):

(1) $$\begin{array}{c} BSM_{i}=\sum_{j=1}^{n}WjPj, \end{array}$$

where BSM = biosecurity score, *W* = weight of the jth biosecurity category, *P* = proportion of practices adopted in each category by dairy farms.

The calculated farm BSM were calculated, as shown in Equations (2) and (3):

(2) $$\begin{array}{c} \text{Weighted external BSM}=\left(\text{livestock purchase and reproduction measures}\times 0.39\right)+\left(\text{transport and carcass removal measures}\times 0.17\right)+\left(\text{feed and water measures}\times 0.1\right)+\left(\text{visitors and farmworkers measures}\times 0.2\right)+\left(\text{vermin and pest control measures}\times 0.14\right), \end{array}$$

(3) $$\begin{array}{c} \text{Weighted internal BSM}=\left(\text{herd health management measures}\times 0.29\right)+\left(\text{calving management measures}\times 0.2\right)+\left(\text{calves management measures}\times 0.21\right)+\left(\text{dairy management measures}\times 0.13\right)+\left(\text{adult cattle management measures}\times 0.07\right)+\left(\text{working organisation and equipment measures}\times 0.1\right). \end{array}$$

Finally, an OLS regression model was developed to explore the overall drivers of the adoption of the weighted BSM, as shown in the formula in Model 2. The independent variables used in the model were farmer education, cattle breed, labour availability, an extra source of income, and value chain choice.

(Model 2) $$\begin{array}{c} Y=\beta 0+\beta 1X1+\beta 2X2+\cdots +\beta pXp+\varepsilon , \end{array}$$

where *Y* is the BSM, *X* are the independent variables (i.e., farmer education, cattle breed, labour availability, an extra source of income, value chain choice, etc.), and *ε* is the error term.

### 2.5. Data Analysis

The survey data were entered into Excel and cleaned and analysed using R statistical software (R statistical software; R Development Core Team, 2020). Descriptive statistics analysis, including means and proportions of adopted measures, were calculated for individual and weighted categories of BSM. OLS regression analysis was undertaken in R statistical software using the lm package.

## 3. Results

### 3.1. Farm Characteristics and Livestock Diseases

Table 1 summarises the demographic characteristics of recruited dairy farms and their survey respondents. The majority of participating farmers were smallholders practising intensive dairy production systems characterised by zero-grazing in small plots of land. The majority of the farmers who participated in this study kept high-producing Holstein-Friesian and their crosses.

Almost all the farmers reported vaccinating their cattle against common diseases such as foot and mouth disease (62.9%), blackleg (78.0%), pasteurellosis (52.2%), anthrax (66.7%), and contagious bovine pleuropneumonia (20.1%) to control the prevalent cattle diseases with high economic impacts.

### 3.2. Adoption of BSM in Smallholder Dairy Farms

Table 2 presents the adoption rates of external BSM adopted by the farms. In the cattle purchasing and reproduction BSM, there was low adoption (below 50%) of requesting proof of origin and health status, testing, and quarantining newly purchased cattle before introducing them to the herd. For the transport of live and dead livestock BSM, only separate transport of purchased cattle had above 50% adoption. There was low adoption of feed and water BSM, particularly low purchase of quality commercial feeds (concentrates) and minerals, low adoption of feed conservation, and just a handful of farms grew their feeds on the farm. The majority of farmers relied on by-products, such as brewers' wastes, hay, and other grain by-products, which are often poorly stored. There was low adoption (below the average of 50%) of sanitation facilities (hand washing or sanitiser) available in the cattle shed and low presence of disinfection foot bath outside the cattle shed in the category of visitors and workers BSM. Finally, regarding vermin and pest control BSM, there was low adoption of controlling overgrown vegetation around cattle sheds, and control plans for insects, rodents, and birds were absent.

Table 3 presents the adoption rates of internal BSM adopted by the farms. There was low adoption of herd health management BSM, including non-physical isolation of sick cattle, lack of hoof disinfection baths in sheds, only a few used dry cow therapy, and the frequent presence of other livestock species and pets on the farm. Regarding calving management BSM, farmers did not isolate or/and test aborting cows and had poor foetal membrane disposal practices. There was a low adoption of housing calves separately in the calves' management BSM. In the dairy management BSM, most farms lacked a separate milking area, and farmers cleaned teats only with water (without a disinfectant) before milking, did not disinfect teats after milking (teat dipping), did not do fore-stripping to check for udder and teat infections (mastitis) and failed to shave the cows' udders and tail. There was low adoption of farm biosecurity plans in the majority of the farms.

### 3.3. Farm-Weighted Biosecurity Scores

Table 4 presents the results of weighted biosecurity scores based on the contribution of various BSM to the overall farm biosecurity. The weighted external biosecurity score was 49.1% which was below average (below 50% adoption), while the weighted internal biosecurity score was 55.5%.

### 3.4. Farm-Level Participant Observations on the Adoption of Biosecurity Measures

Farm-level observations undertaken during the study confirmed the results presented in Tables 2, 3, and *Supplementary 1*. The observations revealed poor ventilation in zero-grazing housing units common in urban areas. Poor housing led to poor animal welfare, with cattle kept tethered with minimum movement, which led to incidences of arthritis and body sores, which were absent in the extensive grazing systems practised in the rural areas. Farmers in these rural areas grazed their cattle in communal grazing fields where different livestock from different farms shared pasture.

Manure disposal was a challenge, especially in urban areas where land sizes per animal capita are smaller and where farmers disposed of manure on the roadsides or in water streams. Just a handful of farmers said they used manure to grow crops on their farmland, e.g., for livestock fodder, and the majority of farmers reported that there was a lack of trade in manure. However, farmers also dried cow dung for use as fuel, while a handful also had biogas plants.

Feed was a challenge in urban areas, and farmers struggled to store large quantities of feed as they were afraid of quality deterioration and contamination. The majority of farmers depended on purchased feeds or grown on rented land. Farmers bought crop residues when the prices were low and stored them as a big heap of teff residues or bales of grass, and they had less control over the quality and safety.

### 3.5. Drivers of the Weighted Farm Biosecurity Score

Farms' overall internal and external biosecurity scores were influenced by farm, demographic, and socio-economic characteristics, as summarised in Table 5. The results of OLS regression show that farmers who had attained secondary and tertiary education were likely to have a high-weighted external and internal biosecurity score. Moreover, participation in both formal and informal milk marketing value chains was likely to lead to a higher internal BSM score.

Farms with a high number of calves had higher external BSM scores. However, farms with the local breed and crosses of exotic breeds had lower external and internal BSM scores.

Farmers who trust the information provided by other farmers also had higher external BSM scores. Additionally, having a veterinarian that used personal protective clothing (PPE) when visiting the farm was likely to lead to a higher internal BSM score. However, trusting government interventions aimed at controlling diseases at the farm level led to a lower BSM score.

Additional availability of male labour was likely to increase the internal BSM score. However, farmers with additional sources of income had significantly lower external and internal BSM scores. Finally, farms that had experienced diseases in the last 2 years had lower external and internal BSM scores.

### 3.6. Drivers of Adoption of Biosecurity Measures (GLMs)

### 3.7. Drivers of Adoption of the Individual Components of Internal and External Biosecurity Measures

The adoption of external BSM was driven by several factors, as summarised in *Supplementary 2*. The breed of cattle, farming system (zero-grazing, semi-intensive, and extensive grazing systems), number of calves, an additional source of income, behaviour of the farm veterinarian, previous experience with a cattle disease in the last 2 years, farmers social networks, choice of milk marketing channel and trust trusted government interventions significantly influenced the adoption of external BSM.

Similarly, the adoption of internal BSM was driven by several factors, as summarised in *Supplementary 3*. The choice of milk marketing channel, farmers' contact with veterinarians, veterinarian behaviour (i.e., the use of PPE), farming system (zero-grazing, semi-intensive and extensive grazing systems), presence of other livestock species in the farm, breed, previous experience to a cattle disease in the last 2 years, farmer's education attainment, an additional source of income, trust on information obtained from other farmers and trust in government interventions aimed at controlling diseases significantly influenced the adoption of internal BSM.

## 4. Discussion

The main objective of this study was to document the current levels of adoption of biosecurity practices in smallholder dairy farms in Ethiopia. Additionally, it aimed at understanding the underlying drivers of biosecurity adoption at the farm level. Prevention and control of endemic pathogens within and between farms depends on the adoption and implementation of BSM [9, 10, 26, 27]. There is a paucity of studies about biosecurity on smallholder dairy farms in Ethiopia, and official or private initiatives for the implementation of biosecurity programmes in the dairy sector are non-existent. The current study provides evidence of low adoption and barriers in the implementation of BSM in smallholder dairy farms in Ethiopia and highlights the measures that can be adopted to control the potential routes of disease introduction and spread of cattle disease within and between farms.

### 4.1. The Current State of Biosecurity Measures Adoption

The adoption of internal and external BSM in smallholder dairy farms in Ethiopia for the prevention of disease transmission, within farms and between farms, was low compared to other studies that have mostly focused on Europe and North America (Tables 2–4) [7, 11, 27–29]. However, the challenge of low adoption of BSM is not unique to smallholder farmers in low and middle-income countries such as Ethiopia but is also a challenge in developed countries in Europe and North America [27–30].

Our study revealed an adoption gap for the BSM assessed (Table 2). External BSM are particularly important for the prevention of disease from external sources [7, 31–33]. Farms purchase of animals without testing, lack of quarantine for purchased cattle, and contact with other cattle during the transport process increases the risk for disease introduction to farms [20]. The major reason for the non-adoption of external BSM in smallholder dairy farms could be due to small farm sizes and the lack of farm space to maintain a physically separated area for quarantine and isolation of purchase or sick livestock [34, 35]. Smallholder farmers in Ethiopia face land scarcity to construct cattle shed, feed storage, isolation, and quarantine areas [36]. Land, which is a key factor in dairy farming, is scarce in Ethiopia [34]. Lack of farm space due to small land parcels has been shown to be a barrier for the adoption of external BSM in Belgium [33]. Moreover, poverty, lack of resources, and absence of a market reward mechanism (i.e., a quality-based payment system for milk) can lead to low adoption of external BSM [34, 37, 38].

The adoption of internal BSM is important to prevent the spread of infectious agents within a farm [31]. Among our study farms, there was low adoption of fundamental herd health management BSM which could increase the chance of disease spreading within the herd (Table 3). Low adoption of footbaths, poor hoof management, and housing have previously been shown to cause hoof overgrowth, lesions between hooves, lesions on legs arthritis, sole ulcers, and lameness in smallholder dairy production in Ethiopia [39]. Lameness and other hoof problems have also been shown to cause high economic losses on dairy farms [32, 39]. Limited or controlled access to cattle sheds, manure storage facilities, and feed storage facilities is recommended to minimise the risk of diseases associated with other livestock and pets (i.e., neosporosis and leptospirosis) [27, 32]. Furthermore, the low use of PPE when handling sick and dead livestock is a public health concern as it could expose farmers to zoonoses [4].

### 4.2. Drivers of Farm Biosecurity Adoption

Results reveal that farmers are not a homogeneous group in the adoption of BSM, and such interventions, to change farmer behaviour must acknowledge the differences in the context of farm sizes, access to capital, and information, among others (Table 4) as has also been recommended by Ritter et al. [26]. The results showed that the farmers' education level is significantly associated with the adoption of internal and external biosecurity practices, which is similar to results that have been reported in other studies. Racicot et al. [40] and Frössling and Nöremark [41] have reported that farmer respondents with higher education had higher biosecurity compliance, and Laanen et al. [30] reported that educated farmers are more knowledgeable about BSM and are convinced about their positive effects.

The adoption of internal and external BSM in our study population was influenced by the demographic and socio-economic characteristics of the farmer, as summarised in Table 5. These results are in agreement with Ritter et al. [26] and Mekonnen et al. [42], who reported that farmers' adoption of BSM was influenced by demographic factors such as age, sex, education, experience, routines, economic, cultural, and the wider social networks influence. Moreover, farmers' social referents (e.g., veterinarians, peers) and governments interventions not only provide technical information but also influence these standards, which is similar to the findings of Ritter et al. [26]. Increasing the adoption and implementation of internal and external BSM requires personal approaches such as individual communication or participatory group learning through extension and veterinarians to enable tailored recommendations that reflect farmers' situations [26].

### 4.3. Policy Implications

In Ethiopia, animal health services are primarily provided by the government, including treatments and vaccinations. Farmers who trusted the government services were less likely to adopt BSM (Table 5). These farmers who trust the government services are likely to be poor and unable to afford to pay for animal health services and unlikely to implement BSM [43]. Government provision of extension and animal health services can act as a financial subsidy or incentive instrument to encourage farmers to implement BSM [12]. A reliance on the government interventions may also create a feeling that it is not up to the farmers to implement preventive measures, which could explain the low adoption of biosecurity practices [43].

Increasing the adoption of BSM requires targeted interventions and policies acknowledging farmers' contextual factors [27]. Sarrazin et al. [7] have argued that farmers bear the direct costs of implementing BSM at the farm level. Moreover, the society, rather than the individual farmer, benefits more from the implementation of BSM, including reduced zoonotic risk, increased international trade, and improved welfare [7].

There is a need for increasing farmers' knowledge regarding the benefits of adopting BSM, including reduction of disease pressure, reduced dependence on antimicrobials, reduced losses associated with cattle illness and mortality, and improved food safety [11, 26, 41, 44]. Farmers prefer to adopt BSM that are easy to implement and with immediate benefits. Farmers are also reluctant to adopt proposed BSM if they are expensive or require resources that are in short supply, including time, labour, or land [45, 46]. Economic incentives such as the implementation of a quality-based milk payment system that rewards farmers who provide high-quality milk with minimal animal disease risks and penalties for poor-quality milk could incentivise farmers to implement BSM [38, 47, 48].

Linking biosecurity and disease control with improving livestock productivity can provide a pathway for sustainable livelihoods' improvements for smallholder dairy farmers [10, 11, 49]. It is to understand that implementation of biosecurity practices suitable for smallholders is not a “one size fits all” and thus is important to acknowledge the geographical, physical, and resource variability in smallholder farming [49]. Although BSM at the herd level reduces the probability of disease introduction into a herd, some measures can be expensive and cumbersome for the farmers [10, 50]. It is therefore important developing BSM recommendations that reflect the context of smallholder farms characteristics to ensure sustained on-farm implementation [10, 11, 50].

## 5. Conclusion

Implementation of BSM at the farm level can improve animal health and welfare, reduce antibiotic use, and increase dairy farm productivity. This study demonstrates that farmers are, to some degree, adopting and implementing BSM, which could contribute to animal, environmental and human health. But given the low adoption of some crucial BSM, the results of this survey may be imperative to enable dairy farmers in Ethiopia to adopt and implement more measures. The low adoption of both internal and external BSM demonstrates the risk of disease introduction and spread between farms and within herds. It is important to understand and acknowledge the individual, cultural, and socio-economic drivers of BSM adoption, to better target interventions and support for farmers. Moreover, there is a need to create an environment that enables farmers to invest and implement BSM, including training and access to extension and capital. Finally, there is a need for proactive engagement with farmers regarding the adoption of BSM, given the known fact that “prevention is better and cheaper than curing diseases.”

## Figures and Tables

**Table 1:** Farm and farmer demographic characteristics (*n* = 159).

	Percentage
Gender	
Male	23.9
Female	76.1
Farmer with an additional source of income	53.5
Dairy farming experience	
<5 years	47.2
5–10 years	14.5
>10 years	38.4
Milk marketing channels (value chains)	
Subsistence (excess milk sold, i.e., beyond household consumption)	23.9
Informal	54.1
Formal	10.1
Both formal and informal	11.9

	**Mean (SE)**

Farmers' age (years)	41.7 (1.0)
Herd sizes	16.5 (1.2)
Number of lactating cows	8.3 (0.7)
Number of calves	11.8 (7.5)
Amount of milk sold per day (litres)	121 (27)
Milk selling price (Ethiopian birr*⁣*^*∗*^/litre)	27 (1)
Labour working at farm	
Adult men working on a farm	3.1 (0.5)
Adult women working on a farm	1.3 (0.2)

S.E., standard error of mean. ^*∗*^Ethiopian birr was equivalent to 0.019 USD in June 2022.

**Table 2:** External biosecurity measures adopted by dairy farmers (in percentage) (*n* = 159).

Purchase and reproduction	%
The farm has a single-controlled entrance	86.2
Farm fenced	91.2
The farm has a farm gate to control movement	96.6
Requires proof of origin and health status when purchasing cattle	13.8
Tests newly purchased cattle for diseases	10.1
Quarantines newly purchased cattle	12.0
Tests milk from purchased cows before introducing them to the herd	8.81
Controls breeding (use of AI) at the farm level	71.1
The mean number adopted out of the eight measures—mean (SD)	3.81 (1.37)

Transport of live and dead livestock	%

Vehicles must pass through a disinfection bath at the entrance	10.1
Purchased cattle transported separately (no shared vehicles)	83.0
Keeps records of cattle deaths	44.0
A post-mortem examination is performed to determine the cause of cattle deaths	12.6
Performs safe disposal of dead cattle carcases	35.2
Farmer uses and cleans PPE used for handling and disposal of dead cattle carcase	32.70
Has dead cattle carcase storage area protected from vermin	47.2
The mean number adopted out of the measures—mean (SD)	2.65 (1.54)

Feed and water	%

Purchase commercial feeds and minerals from markets	4.40
Conserve feeds on farm	47.8
Grow own feeds	30.2
Feeding system (practising intensive rather than extensive grazing systems)	78.0
Feed troughs are used for feeds only (not for other uses)	71.1
Feed troughs cleaned before refilling	99.0
Cattle are given clean and treated water	57.2
Water troughs cleaned before refilling	99.4
The mean number adopted out of the eight measures—mean (SD)	4.83 (1.19)

Visitors and workers	%

Visitors must notify the farmer of their presence	59.1
Sanitation facilities (hand washing or sanitiser) available in the cattle shed	40.9
Employees must wash their hands before entering the cattle shed	60.0
Presence of disinfection foot bath outside cattle shed	28.3
Farmworkers do not work on or visit other cattle farms	75.5
Knows if visitors and workers pose a disease introduction risk (and controls the risk)	59.8
The mean number adopted out of the six measures—mean (SD)	3.25 (1.36)

Vermin and pest control	%

Controls (cuts) overgrown vegetation around the cattle shed	46.5
Has an insect control plan	35.8
Has a rodent control plan	32.7
Has a bird control plan	25.2
Has ectoparasite control plan	76.1
Has an endoparasite control plan	94.3
The mean number adopted out of the six measures—mean (SD)	3.11 (1.52)

PPE, personal protective equipment and clothing; SD, standard deviation.

**Table 3:** Internal biosecurity measures adopted by dairy farmers (*n* = 159).

Herd health management	%
Keeps a register/records of animal health	50.9
Farmer vaccinates dairy cattle when vaccines are available	94.3
Follows a specific vaccination and disease prevention programme	62.0
Physically isolates sick cattle (in a different building away from healthy cows)	28.3
Cows regularly pass through a hoof disinfection baths	14.5
Performs dry cow therapy (DCT)	15.7
No other livestock species on the farm (only cattle present)	59.8
No pet species (dogs and cats) on the farm	30.8
The mean number adopted out of the eight measures—mean (SD)	3.53 (1.43)

Calving management	%

Farm vet performs C-section (for dystocia) (risks contaminating farm)	91.8
Cleans/disinfects cow's hindquarters (before birth)	72.3
Isolate aborted cows	13.8
Test aborted cows to determine causes	9.43
Safe disposal of foetal membranes	44.0
The mean number adopted out of the five measures—mean (SD)	2.31 (0.96)

Calve management	%

Allow calf to suckle colostrum	62.9
Keep calves separated from older cows	91.8
Houses calves individually	46.5
The mean number adopted out of the three measures—mean (SD)	2.01 (0.73)

Dairy management	%

Have a separate milking area	30.2
Clean teats with disinfectant/soap before milking	42.4
Dry teats before milking	92.4
Does fore-stripping check of udder and teat infections (mastitis)	32.1
Disinfects teats after milking (teat dipping)	19.8
Keeps cows upright for a while after milking	56.6
Farmers shave cows' udders and tail	30.2
Farmers discard milk from diseased and treated cattle	69.2
The mean number adopted out of the eight measures—mean (SD)	3.72 (1.82)

Adult cattle management	%

Cattle shed cleaned/disinfected daily	90.6
Groups cows by category (by age, lactation stage, etc.)	69.2
The farmer does daily health checks and monitoring	54.1
Cleans cows' body to remove excrement/dung	95.0
The farm has a biosecurity plan	44.0
The mean number adopted out of the five measures—mean (SD)	3.53 (1.03)

Working organisation and farm equipment	%

Poisonous chemicals are not used or stored in cattle shed	82.9
Uses separate materials for different groups	66.7
Does not share equipment with other farms	100
Medicines and chemicals on the farm are safely stored	93.0
The mean number adopted out of the five measures—mean (SD)	3.42 (0.71)

C-section, caesarean section; SD, standard deviation.

**Table 4:** Weighted biosecurity scores for adopted internal and external biosecurity measures.

Biosecurity measures	Weight (%)	Weighted percentage
Livestock purchase and reproduction	39	18.6
Transport and carcase removal	17	6.4
Feed and water	10	6.0
Visitors and farmworkers	20	7.3
Vermin and pest control	14	10.8
Weighted external biosecurity score		49.1
Herd health management	29	12.8
Calving management	20	9.8
Calve management	21	13.4
Dairy management	13	6.1
Adult cattle management	7	4.9
Working organisation and equipment	10	8.5
Weighted internal biosecurity score		55.5

**Table 5:** Results of generalised linear models of adopted external and internal biosecurity measures scores.

	Weighted external biosecurity score		Weighted internal biosecurity score	
	Coefficient (SE)	*p*-Value	Coefficient (SE)	*p*-Value
(Intercept)	47.9 (6.09)	**<0.001*⁣*^*∗∗∗*^**	54.1 (5.02)	**<0.001*⁣*^*∗∗∗*^**
Farm owner education—primary school	4.07 (4.00)	0.31	5.04 (3.30)	0.13
Farm owner education—secondary school	5.63 (3.97)	0.16	6.67 (3.27)	**0.04*⁣*^*∗*^**
Farm owner education—tertiary school	9.08 (4.16)	**0.03*⁣*^*∗*^**	10.7 (3.43)	**<0.001*⁣*^*∗∗*^**
Marketing value chain—informal	1.86 (2.26)	0.41	0.68 (1.86)	0.72
Marketing value chain—formal	−1.58 (3.51)	0.65	0.84 (2.89)	0.77
Marketing value chain—both formal and informal VC	2.95 (3.44)	0.39	9.16 (2.83)	**<0.001*⁣*^*∗∗*^**
Farmer has additional income	−4.22 (1.97)	**0.03*⁣*^*∗*^**	−3.53 (1.62)	**0.03*⁣*^*∗*^**
Adult males labour	0.22 (0.19)	0.25	0.40 (0.16)	**0.01*⁣*^*∗*^**
Number of calves	0.62 (0.22)	**0.01*⁣*^*∗∗*^**	0.20 (0.18)	0.28
Cattle breed—crosses with exotic breed	−7.95 (2.23)	**<0.001*⁣*^*∗∗∗*^**	−10.18 (1.84)	**<0.001*⁣*^*∗∗∗*^**
Cattle breed—local breeds	−19.3 (4.38)	**<0.001*⁣*^*∗∗∗*^**	−9.99 (3.61)	**<0.001*⁣*^*∗∗*^**
Herd had a disease last 2 years	−4.21 (2.01)	**0.04*⁣*^*∗*^**	−5.30 (1.66)	**<0.001*⁣*^*∗∗*^**
Trusts government interventions	−10.4 (3.67)	**0.01^*∗∗*^**	−4.86 (3.03)	0.11
Trust information from other farmers	8.74 (2.63)	**<0.001^*∗∗*^**	3.15 (2.17)	0.15
Vet uses PPE visiting your farms	4.51 (2.48)	0.07	5.55 (2.05)	**0.01*⁣*^*∗∗*^**
Multiple *R*^2^	0.4089		0.4928	
Adjusted *R*^2^	0.3464		0.4392	

*Note*. Base, farm owner education—no education; marketing value chain—subsistence; cattle breed—exotic breed. VC, value chain; SE, standard error. *⁣*^*∗∗∗*^*P* < 0.001, *⁣*^*∗∗*^*P* < 0.01, *⁣*^*∗*^*P* < 0.05. Bold values signify the significant results.

## Data Availability

The data that support the findings of this study are available from the corresponding author upon reasonable request.

## Additional Points

*Limitation of the Study*. The study may have been biased by the sampling approach (purposive and convenience sampling approach) and the prevailing favourable seasonal conditions, which could have led to the higher milk production per cow compared to the average production data for Ethiopia's farming systems. A previous study has reported an average milk production of 12 l per cow in intensive zero-grazing farms with good feeding practices [51]. Although this study provides a snapshot of BSM adoption in Ethiopia, there is a need of nuanced weighting scores that reflect the animal health risks in smallholder zero-grazing dairy systems. The Biocheck tool was developed in the context of intensive large-scale European dairy farming systems, which may not be fit for the contextual characteristics of smallholder zero-grazing dairy systems. Finally, although this study offers a good insight for what is happening in the urban and peri-urban farms, a follow-up study with a bigger sample size that eliminates observational bias associated with a smaller sample size is needed to better understand the performance of the diverse farming systems present in Ethiopia.

## Supplementary Materials

Supplementary 1 Presents additional information regarding adopted biosecurity practices among sampled dairy farms.

Supplementary 2 Presents the results of generalised linear models of weighted external biosecurity scores, which could explain drivers of the adoption of external biosecurity measures.

Supplementary 3 Presents the results of generalised linear models of weighted internal biosecurity scores, which could explain drivers of the adoption of internal biosecurity measures.
