# Supplementary material for: Adoption of Biosecurity Practices in Smallholder Dairy Farms in Ethiopia
Source: Transbound Emerg Dis. 2023 Aug 14;2023:2277409. doi: 10.1155/2023/2277409 (PMC12016702; doi:10.1155/2023/2277409)
Supplement: Supplementary 1 — Presents additional information regarding adopted biosecurity practices among sampled dairy farms. [file 2277409.f1.docx]

Table S1: Biosecurity practices among sampled dairy farms (n=154)

| The farm has veterinarian contact available and visible to everyone | | 35.8 |
| --- | --- | --- |
| A veterinarian visited within the last 3 months | Planned visit | 26.1 |
|  | Unplanned emergency visit | 65.0 |
| Farm have pets | Dogs | 48.4 |
|  | Cats | 55.3 |
|  | Both cats and dogs | 35.1 |
| Pets (dogs and cats) can access cattle shed |  | 39.6 |
| Dead cattle carcass disposal | Arrange for removal by the municipality | 0.6 |
|  | Burns the dead livestock | 9.4 |
|  | Buries the dead livestock | 21.2 |
|  | Dispose of the dead livestock in the 'bush' | 35.9 |
|  | Fed to other animals in the farm (i.e., dogs) | 25.8 |
|  | Human consumption | 1.9 |
| How farmer disposes of foetal membranes | Dumped into a slurry pit | 1.3 |
|  | They are buried | 25.8 |
|  | They are burned | 15.1 |
|  | They are eaten by the dogs/other animals | 36.5 |
|  | They are put on the manure pile | 1.3 |
|  | Thrown away in the bush | 18.2 |
|  | Thrown into a pit latrine | 1.9 |
| Breeding at farm level | Artificial insemination | 71.1 |
|  | Bull | 10.7 |
|  | Both artificial insemination and bull | 18.2 |
| Feeding system | Extensive | 1.89 |
|  | Semi-intensive | 20.1 |
|  | Intensive | 78.0 |
| Isolates sick animals | Yes, in a physically separated (not shared air volume) | 28.3 |
|  | Yes, physically separated (shared volume of air) | 39.6 |
| Mode of milking | Manual/hand milking | 100.0 |
|  | Machine milking | 0.00 |
| What farmer does with manure | Spread manure on grass/pasture grown for cattle | 20.8 |
|  | Spread manure on grass/pasture grown for cattle | 20.1 |
|  | Discard in rivers/forest and roads | 20.8 |
|  | Fuel (as briquets, dung or biogas) | 31.8 |
|  | Sell manure | 8.4 |
| What farmer does with milk from sick treated cows | Discard it | 69.2 |
|  | Feed to calves | 17.6 |
|  | Home consumption | 2.5 |
|  | Sell it | 10.7 |
| Frequency of ectoparasite control | After more than 3 months | 53.5 |
|  | Every 3 months | 20.8 |
|  | Every month | 1.9 |
| Frequency of endoparasite control | After more than 3 months | 49.7 |
|  | Every 3 months | 40.9 |
|  | Every month | 3.8 |
