# Supplementary material for: Adoption of Biosecurity Practices in Smallholder Dairy Farms in Ethiopia
Source: Transbound Emerg Dis. 2023 Aug 14;2023:2277409. doi: 10.1155/2023/2277409 (PMC12016702; doi:10.1155/2023/2277409)
Supplement: Supplementary 2 — Presents the results of generalised linear models of weighted external biosecurity scores, which could explain drivers of the adoption of external biosecurity measures. [file 2277409.f2.docx]

**Table S2: Results of generalised linear models of weighted adopted external biosecurity scores.**

|  | Purchase and reproduction | | Transport and dead carcass removal | | Feed and water | | Vermin and pest control | | Visitors and workers | |
| --- | --- | --- | --- | --- | --- | --- | --- | --- | --- | --- |
|  | Coefficient  (SE) | Pr(>\|t\|) | Coefficient  (SE) | Pr(>\|t\|) | Coefficient (SE) | Pr(>\|t\|) | Coefficient  (SE) | Pr(>\|t\|) | Coefficient  (SE) | Pr(>\|t\|) |
| (Intercept) | 53.2 (11.29) | 0.00 *** | 33.8 (12.05) | 0.01** | 61.4 (7.53) | 0.00 *** | 44.0 (17.17) | 0.01* | 78.8 (12.12) | 0.00 *** |
| Farm size -medium farms | -2.41 (6.23) | 0.70 | -1.23 (6.65) | 0.85 | -3.55 (4.15) | 0.39 | -4.75 (9.47) | 0.62 | -15.8 (6.68) | **0.02 *** |
| Farm size- large farms | 1.14 (6.53) | 0.86 | -6.94 (6.97) | 0.32 | -3.55 (4.35) | 0.42 | -1.00 (9.93) | 0.92 | -18.2 (7.01) | **0.01*** |
| Farm owner education-primary school | 4.69 (5.95) | 0.43 | 9.02 (6.35) | 0.16 | -2.63 (3.96) | 0.51 | 9.18 (9.04) | 0.31 | 4.13 (6.38) | 0.52 |
| Farm owner education- secondary school | 4.77 (5.98) | 0.43 | 9.19 (6.39) | 0.15 | 2.50 (3.99) | 0.53 | 13.2 (9.10) | 0.15 | 4.22 (6.42) | 0.51 |
| Farm owner education-tertiary school | 7.22 (6.30) | 0.25 | 12.2 (6.73) | 0.07 | 1.29 (4.20) | 0.76 | 13.5 (9.58) | 0.16 | 12.6 (6.76) | 0.06 |
| Farmer has additional income | -5.06 (2.96) | 0.09 | -2.13 (3.16) | 0.50 | -0.77 (1.97) | 0.70 | -4.61 (4.50) | 0.31 | -7.22 (3.17) | **0.02 *** |
| Marketing value chain- informal VC | 5.04 (3.40) | 0.14 | 2.25 (3.62) | 0.54 | 3.06 (2.26) | 0.18 | 3.18 (5.16) | 0.54 | -3.76 (3.64) | 0.30 |
| Marketing value chain- formal VC | 4.46 (5.51) | 0.42 | 1.07 (5.88) | 0.86 | -1.91 (3.67) | 0.60 | -1.15 (8.38) | 0.89 | -8.90 (5.92) | 0.13 |
| Marketing value chain- formal & informal VC | 1.11 (5.18) | 0.83 | -0.94 (5.53) | 0.87 | -8.80 (3.45) | **0.01 *** | 13.3 (7.88) | 0.09 | 4.95 (5.56) | 0.38 |
| Adult males labour | 0.34 (0.31) | 0.28 | 0.54 (0.33) | 0.10 | -0.58 (0.21) | **0.01**** | -0.22 (0.47) | 0.64 | -0.27 (0.33) | 0.41 |
| Calves number | 0.05 (0.38) | 0.89 | 1.11 (0.40) | **0.01 **** | 0.36 (0.25) | 0.16 | 1.03 (0.57) | 0.08 | 0.64 (0.40) | 0.12 |
| Cattle breed - Crosses with exotic breed | -13.48 (3.32) | **0.00 ***** | -7.50 (3.54) | **0.04 *** | 3.60 (2.21) | 0.11 | -6.85 (5.04) | 0.18 | -1.68 (3.56) | 0.64 |
| Cattle breed - Local breeds | -23.68 (6.56) | **0.00 ***** | -8.95 (7.00) | 0.20 | -10.4 (4.37) | **0.02*** | -10.97 (9.98) | 0.27 | -23.0 (7.04) | **0.00 **** |
| Farm system -semi grazing | 0.51 (3.57) | 0.89 | -5.49 (3.81) | 0.15 | -17.6 (2.38) | **0.00 ***** | 7.47 (5.43) | 0.17 | 2.80 (3.83) | 0.47 |
| Farm system -extensive grazing | 10.1 (10.74) | 0.35 | -2.13 (11.47) | 0.85 | -33.2 (7.16) | **0.00 ***** | -3.61 (16.34) | 0.83 | -0.30 (11.53) | 0.98 |
| Herd had a disease last two years | 10.1 (10.74) | 0.35 | -11.2 (3.25) | **0.00***** | -0.19 (2.03) | 0.92 | -8.18 (4.62) | 0.08 | -8.26 (3.26) | **0.01 *** |
| Vet uses PPE visiting your farms | 0.73 (3.04) | 0.81 | 12.7 (4.12) | **0.00 **** | -3.34 (2.57) | 0.20 | 3.20 (5.87) | 0.59 | 7.46 (4.14) | 0.07 |
| Trusts government interventions | -5.28 (5.50) | 0.34 | -8.20 (5.87) | 0.16 | 4.91 (3.67) | 0.18 | -11.6 (8.36) | 0.17 | -20.6 (5.90) | **0.00 ***** |
| Trusts information from other farmers | 5.54 (3.88) | 0.16 | 3.77 (4.14) | 0.36 | 5.84 (2.59) | **0.03 *** | 12.0 (5.91) | **0.04 *** | 13.3 (4.17) | **0.00 **** |
| Farmer group membership | -2.82 (3.46) | 0.42 | -2.41 (3.70) | 0.52 | -4.94 (2.31) | **0.03 *** | -3.89 (5.27) | 0.46 | -1.84 (3.72) | 0.62 |
| Vet services farmer uses -private vet | -4.42 (3.64) | 0.23 | 2.55 (3.89) | 0.51 | -0.69 (2.42) | 0.78 | -1.78 (5.54) | 0.75 | 1.96 (3.91) | 0.62 |
| Vet services farmer uses -private and public vet | -6.07 (3.29) | 0.07 | -3.11 (3.51) | 0.38 | -1.92 (2.19) | 0.38 | 3.28 (5.00) | 0.51 | -9.36 (3.53) | **0.01 **** |
|  |  |  |  |  |  |  |  |  |  |  |
| Multiple R-squared | 0.2484 |  | 0.4796 |  | 0.5452 |  | 0.203 |  | 0.4989 |  |
| Adjusted R-squared: | 0.126 |  | 0.3948 |  | 0.4711 |  | 0.07309 |  | 0.4172 |  |
| p-value: | **0.01**** |  | **0.001 ***** |  | **0.001 ***** |  | 0.06 |  | **0.001 ***** |  |

Base- farm size- small farms, farm owner education-no education, marketing value chain- subsistence, cattle breed – exotic breed, farm system -zero grazing.

VC-value chain, SE- Standard error,*** 0.001, ** 0.01, * 0.05
