# Supplementary material for: Adoption of Biosecurity Practices in Smallholder Dairy Farms in Ethiopia
Source: Transbound Emerg Dis. 2023 Aug 14;2023:2277409. doi: 10.1155/2023/2277409 (PMC12016702; doi:10.1155/2023/2277409)
Supplement: Supplementary 3 — Presents the results of generalised linear models of weighted internal biosecurity scores, which could explain drivers of the adoption of internal biosecurity measures. [file 2277409.f3.docx]

**Table S3: Results of generalised linear models of adopted internal biosecurity measures and the weighted internal biosecurity scores**

|  | Health management | | Calving management | | Calves’ management | | Dairy cow management | | Adult cattle management | | | Equipment and work organisation | |
| --- | --- | --- | --- | --- | --- | --- | --- | --- | --- | --- | --- | --- | --- |
|  | Coefficient  (SE) | Pr(>\|t\|) | Coefficient (SE) | Pr(>\|t\|) | Coefficient  (SE) | Pr(>\|t\|) | Coefficient  (SE) | Pr(>\|t\|) | Coefficient  (SE) | Pr(>\|t\|) | Coefficient  (SE) | | Pr(>\|t\|) |
| (Intercept) | 36.5 (11.1) | 0.00** | 48.3 (13.14) | 0.00 *** | 109.7 (17.8) | 0.00 *** | 21.6 (14.02) | 0.13 | 65.0 (14.0) | 0.00 *** | 110.8 (13.3) | | 0.00 *** |
| Farm owner education-primary school | 7.85 (5.02) | 0.12 | 9.67 (5.94) | 0.11 | 0.39 (8.06) | 0.96 | 11.6 (6.34) | 0.07 | 4.02 (6.31) | 0.53 | -2.97 (6.02) | | 0.62 |
| Farm owner education- secondary school | 4.03 (5.04) | 0.42 | 14.4 (5.96) | **0.02*** | 3.45 (8.10) | 0.67 | 16.0 (6.36) | **0.01*** | 10.1 (6.34) | 0.11 | -5.34 (6.04) | | 0.38 |
| Farm owner education-tertiary school | 10.3 (5.26) | **0.05*** | 15.2 (6.22) | **0.02*** | 11.15 (8.45) | 0.19 | 15.2 (6.64) | **0.02*** | 13.2 (6.61) | **0.05 *** | -1.89 (6.31) | | 0.77 |
| Marketing value chain- informal VC | 1.76 (2.78) | 0.53 | -5.14 (3.30) | 0.12 | 0.67 (4.48) | 0.88 | 9.83 (3.52) | **0.01**** | 3.85 (3.50) | 0.27 | -6.34 (3.34) | | 0.06 |
| Marketing value chain- formal VC | 5.09 (4.42) | 0.25 | -4.44 (5.24) | 0.40 | 1.82 (7.11) | 0.80 | 6.55 (5.59) | 0.24 | 0.17 (5.56) | 0.98 | -1.47 (5.31) | | 0.78 |
| Marketing value chain- formal  and informal VC | 16.8 (4.26) | **0.00 ***** | -2.47 (5.04) | 0.62 | 4.88 (6.85) | 0.48 | 8.86 (5.38) | 0.10 | 13.2 (5.36) | **0.01*** | 1.26 (5.11) | | 0.81 |
| Cattle breed - Crosses with exotic breed | -8.17 (2.83) | **0.00 **** | -11.2 (3.34) | **0.00 **** | -21.8 (4.54) | **0.00 ***** | -7.97 (3.57) | **0.03*** | -4.46 (3.55) | 0.21 | 3.18 (3.39) | | 0.35 |
| Cattle breed - Local breeds | -13.3 (5.43) | **0.02*** | -15.4 (6.42) | **0.02*** | -3.84 (8.72) | 0.66 | -17.47 (6.85) | **0.01 *** | -7.75 (6.83) | 0.26 | 7.36 (6.51) | | 0.26 |
| Farm size medium farms | 1.80 (6.86) | 0.79 | -5.10 (8.12) | 0.53 | -21.9 (11.02) | **0.05 *** | -9.99 (8.66) | 0.25 | -2.07 (8.63) | 0.81 | -5.78 (8.23) | | 0.48 |
| Farm size large farms | -0.78 (7.96) | 0.92 | -5.62 (9.42) | 0.55 | -30.7 (12.80) | **0.02*** | -5.25 (10.05) | 0.60 | -6.16 (10.01) | 0.54 | -4.18 (9.55) | | 0.66 |
| Herd size (cattle in numbers) | 0.26 (0.14) | **0.05*** | 0.00 (0.16) | 0.98 | -0.39 (0.22) | 0.08 | 0.13 (0.17) | 0.46 | 0.14 (0.17) | 0.41 | -0.21 (0.16) | | 0.19 |
| Farmer has additional income | -4.24 (2.45) | 0.09 | -0.70 (2.90) | 0.81 | -7.08 (3.94) | 0.07 | -4.70 (3.10) | 0.13 | -8.34 (3.08) | **0.01**** | 5.17 (2.94) | | 0.08 |
| Other livestock present in farm | -15.8 (2.55) | **0.00 ***** | 2.21 (3.02) | 0.47 | 3.00 (4.10) | 0.47 | 3.92 (3.22) | 0.23 | -4.54 (3.21) | 0.16 | 0.75 (3.06) | | 0.81 |
| Herd had a disease last two years | -5.86 (2.47) | **0.02*** | -10.4 (2.92) | **0.00 ***** | -3.40 (3.97) | 0.39 | -5.21 (3.12) | 0.10 | -5.21 (3.10) | 0.10 | 1.90 (2.96) | | 0.52 |
| Vet contacted you about vaccination programmes | 12.5 (2.91) | **0.00 ***** | -1.23 (3.44) | 0.72 | -6.42 (4.67) | 0.17 | 15.5 (3.67) | **0.00 ***** | 7.36 (3.66) | **0.05 *** | -7.76 (3.49) | | **0.03 *** |
| Veterinarian uses PPE at your farms | 0.66 (3.20) | 0.84 | 11.0 (3.79) | **0.00 **** | 4.96 (5.14) | 0.34 | 12.4 (4.04) | **0.00 **** | 9.42 (4.02) | **0.02*** | -9.53 (3.84) | | **0.01 *** |
| Trusts government interventions | 1.50 (4.51) | 0.74 | -7.51 (5.34) | 0.16 | 1.02 (7.25) | 0.89 | -13.4 (5.70) | **0.02 *** | -9.27 (5.67) | 0.10 | -0.81 (5.41) | | 0.88 |
| Trust information other farmers | -1.19 (3.25) | 0.72 | 7.54 (3.85) | 0.05 | 1.25 (5.23) | 0.81 | 10.3 (4.11) | **0.01 *** | 2.05 (4.09) | 0.62 | -2.60 (3.91) | | 0.51 |
|  |  |  |  |  |  |  |  |  |  |  |  | |  |
| Multiple R-squared | 0.48 |  | 0.3678 |  | 0.2722 |  | 0.4955 |  | 0.3786 |  | 0.2551 | |  |
| Adjusted R-squared: | 0.4126 |  | 0.2859 |  | 0.178 |  | 0.4301 |  | 0.2981 |  | 0.1586 | |  |
| p-value: | **0.01 **** |  | **0.01 **** |  | **0.01 **** |  | **0.01 **** |  | **0.01 **** |  | **0.01 **** | |  |

Base- farm size- large farms, farm owner education-no education, marketing value chain- subsistence, cattle breed – exotic breed, farm system - zero-grazing.

VC-value chain, SE- Standard error,*** 0.001, ** 0.01, * 0.05
